# Dietary Habits of Individuals With Primary Sclerosing Cholangitis—Poor Fat‐Soluble Vitamin Intake and Dietary Quality

**DOI:** 10.1111/liv.16182

**Published:** 2024-11-27

**Authors:** Catarina Lindqvist, Michael Ingre, Stergios Kechagias, Emma Nilsson, Antonio Molinaro, Fredrik Rorsman, Annika Bergquist

**Affiliations:** ^1^ Section Clinical Nutrition Karolinska University Hospital Stockholm Sweden; ^2^ Unit of Gastroenterology and Hepatology, Department of Medicine Huddinge Karolinska Institutet Stockholm Sweden; ^3^ Department of Health, Medical, and Caring Sciences Linköping University Linköping Sweden; ^4^ Deparment of Surgery and Gastroenterology Skåne University Hospital Lund/Malmö Sweden; ^5^ Wallenberg Laboratory, Department of Molecular and Clinical Medicine University of Gothenburg Gothenburg Sweden; ^6^ Department of Gastroenterology and Hepatology Uppsala University Hospital Uppsala Sweden; ^7^ Department of Upper Abdominal Diseases Karolinska University Hospital Stockholm Sweden

**Keywords:** cholestatic liver disease, dietary intake, micronutrients, nutrition, nutritional assessment

## Abstract

**Background and Aims:**

Individuals with primary sclerosing cholangitis (PSC) have expressed a need for more dietary information. The aim of this study was to evaluate the dietary intake of individuals with PSC and compare it with Nordic nutrition recommendations 2023 (NNR2023).

**Methods:**

A cross‐sectional assessment of dietary intake was performed using a food‐frequency questionnaire among 120 individuals with PSC from five regions across Sweden. Macro‐ and micronutrient intake was compared to NNR2023. Dietary quality was evaluated using an index developed by the National Food Agency in Sweden.

**Results:**

The median age was 47 years (IQR 18), and median body mass index (BMI) was 25.2 kg/m^2^ (IQR 5.9). Eight percent had a BMI < 20, and 13% had a BMI > 30. The average fibre intake was 18 g (IQR 18). Median energy distribution included 36% from fat (15% saturated, 4.6% polyunsaturated), 17% from protein and 43% from carbohydrates, highlighting an imbalanced diet with low carbohydrate, fibre and polyunsaturated fat intake and high saturated fat consumption. More than half reported suboptimal intake of zinc, selenium and vitamins C, D and K and > 30% suboptimal intake of vitamins A, B6, E, niacin, folate, potassium, magnesium and iron. Forty percent had poor dietary quality. Longer PSC duration and previous colectomy were associated with a lower dietary quality.

**Conclusions:**

Many individuals with PSC do not reach the recommended levels of various micronutrients, especially fat‐soluble vitamins and report a poor dietary quality. The results highlight the need for a comprehensive approach to nutritional management in this population.

**Trial Registration:**

ClinicalTrials.gov identifier: NCT04133792

**Summary**

- Individuals with primary sclerosing cholangitis (PSC) often lack proper dietary guidance.
- This study found that many individuals with PSC report an imbalanced diet, with low intake of carbohydrates, fibre and polyunsaturated fats, and high intake of saturated fats.
- Additionally, more than half reported insufficient intake of important vitamins and minerals, particularly fat‐soluble vitamins.
- Overall, many participants reported poor dietary quality, suggesting a need for better nutritional management for individuals with PSC.

**Abbreviations**
BMI: body mass index
IBD: inflammatory bowel disease
MASLD: metabolic‐associated steatotic liver disease
NNR: Nordic nutrition recommendations
PSC: primary sclerosing cholangitis

## 1 Introduction

PSC is a chronic cholestatic liver disease marked by inflammation in bile ducts and often coexists with inflammatory bowel disease (IBD) (70%) [1]. There is a high risk of liver failure, cholangiocarcinoma, need for liver transplantation, or death within 15–20 years after diagnosis. Disease progression varies from early stages, where patients may feel generally healthy or fatigued, to advanced stages with symptoms like jaundice, abdominal pain, weight loss and vitamin deficiencies [2, 3]. Geographical incidence varies, notably high in northern Europe, making PSC the primary reason for liver transplantation in Nordic Countries [4]. Alterations in the intestinal microbiome may play a role in the pathogenesis of both IBD and PSC, although previous studies show associations and not a causal pathway [5]. Dietary habits, potentially impacting the human microbiome [6], are speculated to be relevant to PSC pathogenesis, yet their influence on the disease remains unclear. It remains unknown if dietary patterns affect the progression of PSC, and descriptions of dietary intake in patients with PSC are scarce. Being diagnosed with a chronic progressive illness might influence lifestyle and food habits. In addition, concurrent chronic conditions such as IBD can also exacerbate the risk [7]. One previous North American study on individuals with PSC revealed differences in dietary intake and food preparation methods compared to controls, highlighting unique patterns like reduced fish consumption and a preference for more well‐done steak/burgers [8]. However, such patterns may differ in Europe which may have some differences in food culture. In a recent study on patient‐reported quality of care in PSC, half of the respondents expressed a desire for more information on food, diet and supplements [9]. This emphasises the importance of investigating dietary factors and possible associations between diet and disease progression. Living with a disease like PSC, which carries a risk of malabsorption and malnutrition as it progresses [10], may increase the need for a high‐quality diet that meets the nutritional requirements for energy, vitamins and minerals. The overall aim of this study is to describe and evaluate dietary intake in patients with PSC and its correlation with disease severity. Specifically, the study investigates whether the diets of individuals with PSC, at a group level, meet average requirements for micronutrients and fibre. This will provide a basis for assessing the nutritional adequacy within this population.

## 2 Materials and Methods

### 2.1 Study Design and Population

The data collection in this cross‐sectional study was performed at baseline in patients included in PiSCATIN study, which is an ongoing, randomised placebo‐controlled multicentre study in Sweden where patients with PSC will be included and randomised to daily intake of 40 mg simvastatin or placebo for 5 years [11]. A full list of inclusion and exclusion criteria can be find elsewhere [11]. Briefly, inclusion criteria is a confirmed PSC diagnosis based on EASL guidelines [12]; exclusion criteria are patients listed for liver transplantation, previous liver transplantation or having advanced end‐stage liver disease (Child‐Pugh score > B9 points). Individuals from five of the 11 recruiting centres were asked to participate in this study.

### 2.2 Clinical Variables

Clinical information was collected as part of the PiSCATIN study, including variables regarding age, gender, weight, height, BMI, co‐morbidities such as IBD, diabetes and colectomy. Liver stiffness was measured by elastography (Fibroscan, Echosens, Paris, France). Advanced liver disease was defined as presences of clinical/imaging signs of portal hypertension (enlarged spleen and/or varices and/or ascites) or liver stiffness ≥ 15 k Pascals (kPa) at fasting elastography examination. The presence of liver‐related symptoms such as pruritus, fatigue, right upper quadrant pain, jaundice that required intervention or endoscopic retrograde cholangiopancreatography (ERCP) or bacterial cholangitis treated with antibiotics during the latest 12 months was reported.

### 2.3 Dietary Intake

Dietary intake was assessed through MEAL‐Q, a web‐based food‐frequency questionnaire (FFQ), within 6 months of the baseline examinations. MEAL‐Q was developed by researchers at Karolinska Institutet and has been validated against a web‐based, 7‐day weighed food diary for macro‐ and micronutrients [13, 14]. MEAL‐Q has a meal‐based and interactive format, that is, only those who report consumption of a certain food will receive follow‐up questions related to details of this consumption. The FFQ was designed to capture dietary habits during the previous 2 months and took approximately 10–20 min to complete. Intake of macro‐ and micronutrients was calculated based on the reported intake frequencies and portion sizes reported in the Meal‐Q. The nutrient conversions did not include dietary supplements. The nutritional calculations were done with an algorithm developed by the research group that developed the questionnaires [13, 14]. Macro‐ and micronutrient intake was compared to the recently published Nordic Nutrition Recommendations (NNR) 2023 [15], and sex‐ and age‐adjusted levels were used when applicable. The average requirement was used to compare the reported intake of levels of micronutrition, average requirement is the average daily nutrient intake level that is estimated to meet the requirements of half of the individuals in a particular life‐stage group in the general population [15]. The average requirement is usually used to assess the adequacy of nutrient intake of groups of people. For some vitamins where average requirement was not available, provisional average requirement was used. Provisional average requirement approximates average requirement, and has larger uncertainty than average requirement. It is calculated by multiplying average requirement by a factor of 0.8 [16]. An intake lower than the provisional average requirement on the group level does not necessarily point to inadequacy [15].

A dietary quality index developed by the National Food Agency in Sweden was used to determine dietary quality [17]. The index is designed to capture dietary quality (fibre, fat and discretionary foods) and compliance with dietary recommendations (intakes of fruit, vegetables, fish and whole grain bread). It scores intake of fruit and vegetables, whole grain bread, fish and shellfish, discretionary foods (sweets, cakes, soft drinks and French fries), margarine and butter, cheese and sausage, with a total min–max score of 0–12 points. Dietary quality was defined as poor (< 4 points), fair (5–8 points) or high (> 9 points). Positive contributors to the index are frequent intakes of fruit, vegetables, whole grain bread and fish. Negative contributors are frequent intakes of cheese, sausage and discretionary foods. The use of low‐fat margarine (< 40%) contributes positively while the use of high‐fat margarine or butter (> 60%) contributes negatively.

### 2.4 Statistical Analysis

Categorical data are reported as number and percentages, and differences between groups were analysed using the Chi‐2 test. Continuous data are reported as medians and interquartile ranges and analysed using the Kruskal–Wallis test or independent t‐test. Patients with missing data were not included in the analysis for that missing covariate. Participants were grouped into mild PSC (no liver‐related symptom), or symptomatic PSC which was defined as one or more liver‐related symptoms.

To investigate which factors were related to dietary quality, a set of linear regression analyses was performed with dietary quality total score as the dependent variable, and age, gender, PSC duration, severity of PSC, liver symptoms, IBD diagnosis and colectomy as independent variables. The analysis was performed in three steps: first, a set of univariable models was fit between the dependent and all independent variables for descriptive purposes. The second step was to include all independent variables in a multivariable model and in a final step backward elimination was used to reduce the complexity of the model and only keep the most important variables. Sensitivity analyses were performed to test for a potential interaction age*gender and for a curvilinear effect of age using natural splines with 2°–3° of freedom, but no improvement on Akaike Information Criterion was found over the final model. *p* < 0.05 was considered statistically significant. The statistical analyses were performed with SPSS version 27 (IBM Corp., Armonk, NY, USA) and with R 4.1.0 statistical software.

### 2.5 Ethical Considerations

Informed written consent was obtained from all patients. This study was conducted in accordance with the Helsinki Declaration and was approved by the Swedish Ethical Review Authority 2018/2462‐31, Dnr 2019‐00611, 2020‐03414 and 2020‐00471 and by the Swedish Medical Products Agency (EudraCT 2018‐200 814‐39). The ClinicalTrials.gov identifier for PiSCATIN is NCT04133792.

## 3 Results

**FIGURE 1:**
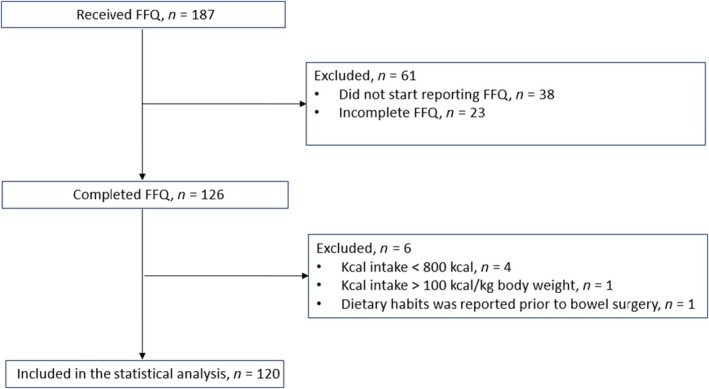
Flow chart of the study. FFQ, food frequency questionnaire, n; number, kcal; kilocalories, kg; kilogram.

A total of 126/187 participants completed the FFQ between November 2020 and January 2023, which equals to a response rate of 67.4%. Six participants were excluded from the analysis: one female with a reported high energy intake which was deemed unplausible with a reported intake of 105 kcal/kg body weight, four male individuals with a reported intake < 800 kcal/day and one individual who reported dietary intake as it was before bowel surgery leaving in total 120 individuals in the analysis. See flowchart for a description of the inclusion and exclusion of participants (Figure 1). The clinical characteristics of the study cohort are shown in Table 1. The median age was 47 years (interquartile range, IQR 18) and the majority, 90 (73%), were male. The median body mass index (BMI) was 25.2 (IQR 5.9), 10 persons (8%) had a BMI < 20 and 16 (13%) had a BMI > 30. The average duration of PSC was 8.6 years (IQR 10.2). Around half of the subjects (48%) had liver‐related symptoms. Liver stiffness above 15 kPa, was found in 17%.

**TABLE 1:** Patient characteristics in 120 individuals included in the study.

Variable	*N*	
Sex, male	120	87 (73%)
Age, years (IQR, Q1‐Q3)	120	47 (18, 36–54)
Weight, kg	120	79 (18, 71–89)
BMI, kg/m^2^ (IQR, Q1‐Q3)	120	25.2 (5.9, 22.4–28.3)
< 20		10 (8%)
20–25		49 (41%)
25–30		45 (38%)
> 30		16 (13%)
Duration of PSC, years	118	8.6 (10.2), 4.6–14.7
Liver stiffness, elastography, kPA (IQR, Q1‐Q3)	114	7.8 (5.8, 5.5–11.3)
< 5		21 (18%)
5–10		60 (53%)
10–15		14 (12)
15–20		6 (5)
> 20		13 (11)
Comorbidity at baseline, yes/no		
IBD	120	92 (77%)
Diabetes	120	2 (2%)
Colectomy	120	17 (14%)
FIB‐4 score (IQR, Q1‐Q3)	118	1.05 (0.79, 0.79–1.6)
INR (IQR, Q1‐Q3)	119	1.0 (0.2, 0.9–1.1)
Bilirubin, μmol/L (IQR, Q1‐Q3)	120	12 (9.2, 8.6–17.8)
Albumin, g/L (IQR, Q1‐Q3)	120	38.0 (4, 36.0–40.0)
CRP, mg/L (IQR, Q1‐Q3)	120	2.0 (3.0, 1.0–4.0)
ALP, U/L (IQR, Q1‐Q3)	120	135 (125, 157–204)
Lipids (IQR, Q1‐Q3)		
HDL, mmol/L	116	1.7 (0.7, 1.3–2.0)
LDL, mmol/L	116	3.0 (1.2, 2.3–3.5)
TG, mmol/L	116	0.9 (0.6, 0.7–1.3)
Symptomatic PSC^†^	120	57 (48%)
Severe PSC^‡^	112	40 (36%)

Abbreviations: ALP, alkaline phosphatase; BMI, body mass index; CRP, c reactive protein; HDL, high‐density lipoprotein; IBD, inflammatory bowel disease; INR, international normalised ratio; kg, kilogram; kPA, kilopascal; LDL, low‐density lipoprotein; PSC, primary sclerosing cholangitis; TG, triglycerides.

^†^ Symptomatic PSC defined as one or more liver‐related symptoms (pruritus, fatigue, right upper quadrant pain, jaundice that required intervention or ERCP during the latest 12 months or bacterial cholangitis treated with antibiotics during the latest 12 months).

^‡^ Severe PSC defined as liver stiffness > 15 kPA and/or current varices and/or splenomegaly.

### 3.1 Macro‐ and Micronutrients

The daily energy intake was 1762 (IQR 1132) kcal (95% CI 1759–2067)). Percentage of energy from fat, saturated fat, polyunsaturated fat, protein and carbohydrate were 36%, 15%, 4.6%, 17% and 43% respectively (Table 2). Mean intake for dietary fibre was 18 g/day, which is below the recommended intake of 25–35 g/d for both men and women [15]. PSC‐IBD individuals had a higher intake of total fat (36.8 (IQR 7) vs. 33.8 (SD 8) E%, *p* = 0.025) and saturated fat (14.9 (IQR 4) vs. 13.6 (IQR 4) E%, *p* = 0.018). There was a tendency for a lower carbohydrate intake in PSC‐IBD individuals (42.5 E% (IQR 6) vs. 45.2 E% (IQR 10) (*p* = 0.095). No differences were seen in macronutrient intake when comparing individuals with symptomatic disease versus individuals with nonsymptomatic disease.

**TABLE 2:** Reported energy intake and intake of macronutrients in the study population.

	Average intake NNR 2023^a^	All (*n* = 120)	Non‐IBD (*n* = 28)	PSC‐IBD (*n* = 92)	*p*‐value
Energy, MJ	6.5–11.2 MJ/d	7.4 (4.7), 7.4–8.7	7.2 (4.5), 6.3–9.0	7.4 (4.7, 7.4–8.9	0.409
Energy, kcal		1762 (1132), 1759–2067	1710 (1087), 1507–2160	1771 (1132), 1759–2114	0.409

Macronutrient	Recommended intake NNR 2023	All (*n* = 120)	Non‐IBD (*n* = 28)	PSC‐IBD (*n* = 92)	*p*‐value
Carbohydrates, E%	45–60	43.3 (7), 41.8–44.1	45.2 (10), 41.8–46.9	42.5 (6), 41.2–43.8	0.095
Dietary fibres, gram	25–35	18 (12), 18–23	17 (12), 17–31	18 (11), 18–22	0.877
Protein, E%	10–20	16.9 (3), 16.6–17.6	17.2 (3), 15.9–18.4	16.8 (4), 16.5–17.7	0.960
Total fat, E%	25–40	36.4 (7), 35.4–37.4	33.8 (8), 32.6–36.6	36.8 (7), 35.8–36.9	**0.025**
Saturated fat E%	< 10	14.8 (4), 14.8–16.1	13.6 (4), 13.0–15.5	14.9 (4), 15.0–16.6	**0.018**
Monounsaturated fat E%	10–20	12.8 (2), 12.5–13.2	12.4 (3), 11.5–13.4	13.0 (2), 12.6–13.4	0.203
Polyunsaturated fat, sum E%	5–10	4.6 (2), 4.7–5.3	4.7 (2), 4.4–5.6	4.6 (2), 4.7–5.4	0.975
Omega‐6 polyunsaturated fat E%	> 3	3.7 (2.6), 3.8–4.6	3.7 (0.2), 3.5–4.5	3.5 (2.0), 3.5–4.2	0.646
Omega‐3 polyunsaturated fat E%	> 1	0.9 (0.5), 0.9–1.1	0.8 (0.0), 0.8–1.0	0.9 (0.0), 0.9–1.0	0.362

*Note:* Median (IQR), 95% CI are presented. P value less than 0.05 are marked with bold font, indicating significance.

Abbreviations: CI, confident intervals; E%, energy percentage; IQR, interquartile range; *N*, number; NNR, Nordic nutrition recommendations; RI, recommended daily intake.

^a^ Average energy intake ranges from 6.5 to 11.2 MJ/d in healthy adults [15]. Kruskal–Wallis test.

Twenty‐six percent of participants reported regular consumption of vitamins, minerals or other supplements, whereas 20% indicated occasional intake, and 51% reporting never doing so. Among the supplements, multivitamins with minerals were the most reported (13% of participants reported daily intake and 15% weekly or less regular), followed by daily consumption of vitamin D by 11%. Two percent reported daily supplementation of vitamins A and E.

### 3.2 Alcohol Consumption

Alcohol consumption in units was reported between a minimum of 0 to a maximum of 11.5 units/week (Figure S1). The median reported intake was 1.4 (IQR 3.5) units/week. Individuals with fair to high dietary quality reported a median alcohol intake of 0.8 units per week (IQR 2.9), while those with a poor dietary quality index reported a median intake of 1.8 units per week (IQR 4.6) (*p* = 0.05).

Most participants (63%) reported consumption of alcoholic beverages at least once per month. Sixty‐two (49%) individuals reported drinking wine at least once per month, and 14 (12%) reported a consumption of three or more bottles of wine per month. Beer was consumed by 41%, spirits by 16% and cider by 5%.

### 3.3 Comparing Micronutrient Intake to the Estimated Average Requirements

**FIGURE 2:**
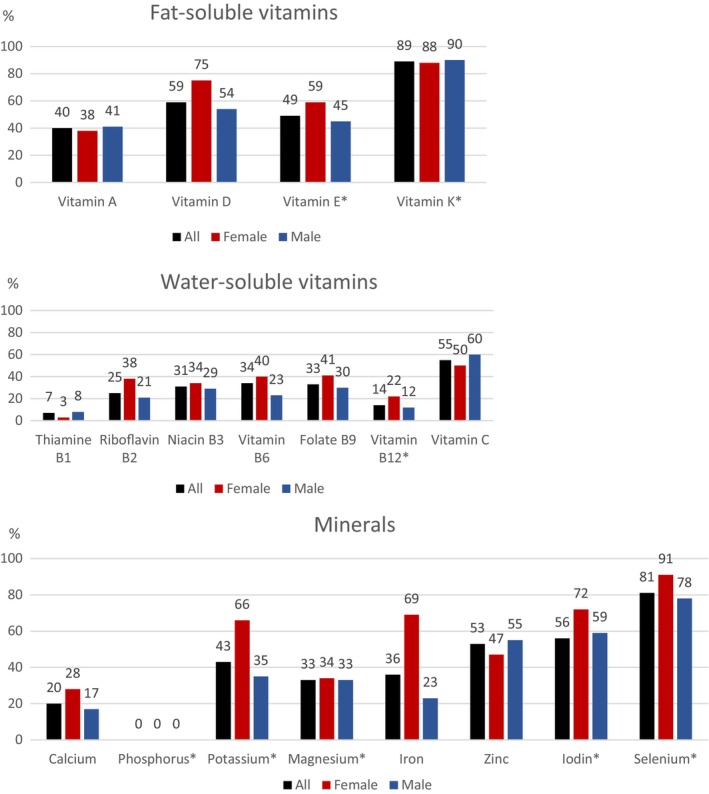
Percentage of the PSC study cohort with a reported intake below average requirement of micronutrients. Figure shows how large part of the population in percentage reported an intake of vitamins/minerals below the average requirement according to the Nordic Nutrition Recommendations 2023 [15]. Age‐and gender adjusted average requirements have been used for the micronutrients when suitable. *Micronutrients compared to provisional average requirements which has a higher uncertainty.

Inadequate intake for vitamins C, D and K was reported by more than 50% for both sexes (Figure 2 and Table S1). In addition, greater than 30% reported suboptimal intakes of other micronutrients such as vitamins A, B6, E, niacin and folate. For vitamin D, 59% reported intakes below the average requirement of 7.5 μg/day [15]. For vitamin C, 55% reported intakes below the average requirements of 75/90 mg/day. Eighty‐nine percent reported vitamin K intakes below the provisional average requirement of 50/60 μg/day respectively. Forty percent reported intakes of vitamin A below the average requirement of 540/640 retinol equivalents/day. For vitamin E, 49% reported intakes below the provisional average requirement of 8/9 α‐tocopherol/day.

More than 50% of the subjects had suboptimal intake of zinc, iodin and selenium. In addition, greater than 30% had suboptimal intakes of potassium, magnesium and iron. For iron, 69% of women and 23% of men reported intakes below the average requirement of 9/7 mg/day.

No differences were seen in micronutrient intake when comparing PSC‐IBD with non‐IBD (Table S2).

### 3.4 Dietary Quality Index

**FIGURE 3:**
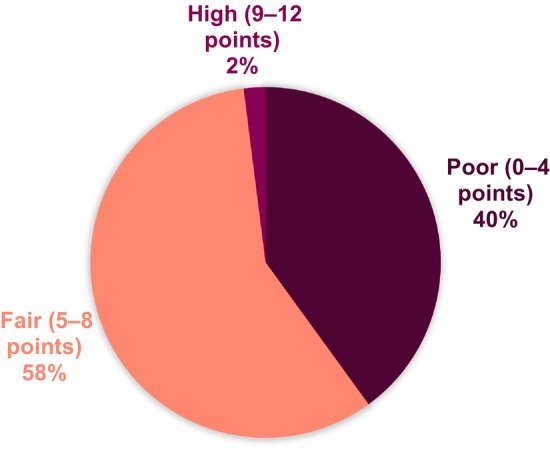
Dietary quality in individuals with primary sclerosing cholangitis in the study cohort. Dietary quality was defined as poor (≤ 4 points), fair (5–8 points) or high (> 9 points). Positive contributors to the index are frequent intakes of fruit, vegetables, whole grain bread and fish. Negative contributors are frequent intakes of cheese, sausage and discretionary foods.

**FIGURE 4:**
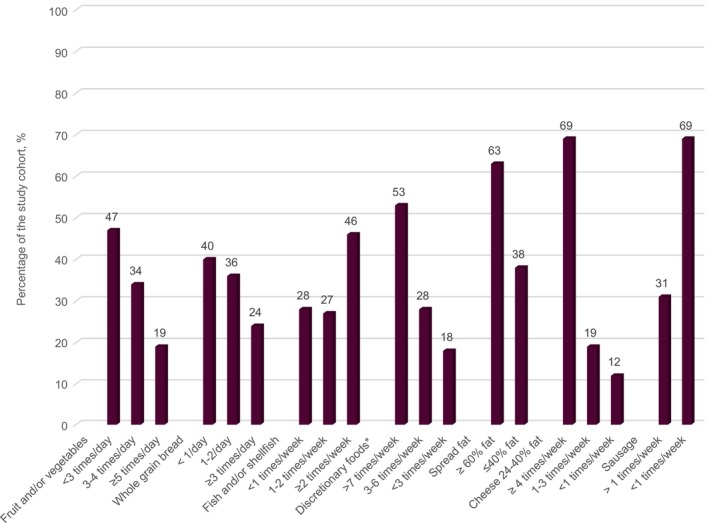
Frequency of reported intake of different food items in the study cohorts. *Candy, cakes, soda/squash, fries etc.

The median dietary index score was 5.0 (95% CI 4.6–5.2) points, ranging from 1 (poor quality) to 10 (high quality). Dietary quality was classified as fair for 58% of the participants, while 40% had poor dietary quality and 2% had high‐quality diets (Figure 3). Forty‐seven percent reported consuming fruit and vegetables < 3 times/day, and 40% reported consuming whole grain bread < 1/day. Twenty‐eight percent of the population reported to eat fish and shellfish < 1 time/week. Discretionary foods such as candy, cake, soda and fries were consumed > 7 times/week by 53% of the responders (Figure 4).

To investigate what factors in PSC that was related to the dietary quality we performed a linear regression analysis. The results presented in Table 3 show that three factors were related to dietary quality and were included in the final model. Older age increased dietary quality with 0.32 (± 0.14, standard error, SE) points per 10 years, but longer duration of PSC decreased dietary quality by almost twice that amount, or −0.62 (SE ± 0.23) points per 10 years, and having a colectomy reduced dietary quality by −1.1 (SE ± 0.47) points (comparable to 17.8 years of PSC duration). In the multivariable model only PSC duration was statistically significant at *p* < 0.05 and in the univariable model PSC duration and colectomy, but not age, reached significance (Table 3). There were no statistically significant differences in albumin (*p* = 0.914) and triglycerides (*p* = 0.187) between the groups with low dietary quality and those with fair and high dietary quality.

**TABLE 3:** Factors related to dietary quality index in the study population. Linear regression.

	Univariable		Multivariable		Backwards elimination	
Variable	*b* ± se	*p*	*b* ± se	*p*	*b* ± se	*p*
Age	0.28 ± 0.15	0.063	0.29 ± 0.15	0.065	0.32 ± 0.14	**0.028**
Gender	0.53 ± 0.38	0.166	0.20 ± 0.38	0.610		
PSC duration	−0.60 ± 0.23	**0.012**	−0.58 ± 0.24	**0.017**	−0.62 ± 0.23	**0.008**
Liver‐related symptoms	0.58 ± 0.35	0.095	0.32 ± 0.36	0.378		
Severe PSC	−0.21 ± 0.36	0.562	0.04 ± 0.39	0.919		
IBD	−0.85 ± 0.41	**0.042**	−0.49 ± 0.42	0.242		
Colectomy	−1.19 ± 0.48	**0.014**	−0.91 ± 0.49	0.066	−1.10 ± 0.47	**0.021**

*Note: b* ± se = coefficient b. P value less than 0.05 are marked with bold font, indicating significance.

Abbreviations: CI, confidence interval; IBD, inflammatory bowel disease; PSC, primary sclerosing cholangitis; se, standard error.

## 4 Discussion

The results of this study show that individuals with PSC report an imbalanced diet, with low intake of carbohydrates, fibre and polyunsaturated fats, and high intake of saturated fats. Additionally, more than half of the subjects reported suboptimal intake of the micronutrients zinc, iodine, selenium and vitamins C, D and K, and over 30% suboptimal intakes of vitamins A, B6, E, niacin, folate, potassium, magnesium and iron.

Many participants reported low vitamin D intake. In PSC with chronic cholestasis, malabsorption is frequent [10]. Low intake of fat‐soluble vitamins was commonly reported and combined with malabsorption that poses a risk of developing deficiency. Similar findings are observed in populations with IBD, where vitamin D intake is significantly lower than in controls [18]. Over 50% reported low intake levels of vitamin D and only 11% reported daily supplementation. Severe vitamin D deficiency at PSC diagnosis and persistent deficiency are linked to an increased risk of hepatobiliary malignancies, liver‐related mortality or the need for transplantation [19]. Cholestasis is associated with an increased risk of bone mass loss and osteoporosis [20, 21], which low vitamin D intake may exacerbate. Forty percent reported low vitamin A intake, highlighting the need for conducting deficiency testing and supplementation. Vitamin A deficiency may affect vision, immune function and is linked to liver fibrosis and liver‐related mortality [22, 23, 24].

Recent studies highlight the importance of vitamin B6 in PSC. One study found that vitamin B6 deficiency is common in PSC and is linked to shorter liver transplantation‐free survival. [25]. In this study, 34% of participants had vitamin B6 intake below the average requirement, which is lower than the general Swedish population [26]. This suggests that individuals with PSC consume less vitamin B6‐rich food or smaller portions. Dietary advice should consider vitamin B6 intake.

A low fibre intake was reported. Previous research indicates that individuals with IBD often perceive high fibre foods like fruits and vegetables as triggers for relapse or worsened symptoms, leading to low fibre intake in [27, 28, 29, 30]. We found no difference in fibre intake between those with or without IBD, and low fibre intake is also noted in the general Swedish population [26].

The average dietary quality was fair, slightly lower than the general Swedish population in 2008 [17]. A Swedish study on individuals with rheumatoid arthritis from 2018 found that only 15% had a poor diet [31], compared to 40% in our current study. It should be noted that there has been no recent investigation into dietary habits among the general Swedish population in the past 15 years. Comparison of the dietary intake of PSC patients with that of healthy individuals may not be appropriate due to the unique nutritional challenges faced by individuals with PSC. These challenges, such as PSC‐related abdominal pain, nausea and fatigue, malabsorption and the need for specific dietary adjustments, may affect their dietary choices. Additionally, the frequent coexistence of IBD in PSC patients may further complicate their nutritional habits. These factors together can influence dietary intake, making direct comparisons with healthy individuals misleading. We found no association between dietary quality and the presence of IBD or symptoms. Only PSC duration and previous colectomy were independently associated with lower dietary quality, while higher age was associated with better dietary quality. This suggests that dietary habits are influenced by factors other than PSC‐related symptoms or comorbidities in this population.

Some limitations should be acknowledged, such as the cross‐sectional design, the use of a food frequency questionnaire which is dependent on self‐reported data, no available laboratory values on vitamin status and the absence of a control group. Therefore, potential causal relationships between diet and the severity of PSC cannot be found and results need to be cautiously interpreted. Recall bias cannot be ruled out. Investigating dietary intake has methodological challenges. FFQ was chosen over other dietary assessment methods due to its ability to capture long‐term dietary patterns. FFQs are superior to 24‐h recall or 3‐day food diaries at estimating intake of foods that are consumed episodically, as they capture frequency and portion size over a longer period [32]. The used FFQ is validated and shows good agreement compared to the gold standard, a 7‐day weighed diary [14]. Another limitation is the absence of biomarkers to assess vitamin and mineral status. Triglycerides and albumin were available, but no associations were found between these biomarkers and dietary quality.

The study underscores the need for further research and targeted dietary interventions for individuals with PSC. These findings can inform health promotion programmes that emphasise polyunsaturated fats and essential micronutrients. Many centres delay dietary assessment and counselling until malnutrition or severe cholestasis occurs, but early intervention could be beneficial. Physicians should consider routine assessments of micronutrient deficiencies in their practice.

In conclusion, the presented results offer new insights into the dietary habits of individuals with PSC. A significant portion reported suboptimal intake levels of polyunsaturated fats and several micronutrients, particularly fat‐soluble vitamins. Additionally, over a third of the population reported insufficient intake of vitamin B6, which could potentially impact long‐term prognosis if not addressed. These imbalances in both macronutrient and micronutrient intake underscore the necessity for a comprehensive approach to nutritional management in this population.

## Author Contributions

Study conception and design: Catarina Lindqvist and Annika Bergquist. Acquisition of data: Catarina Lindqvist, Stergios Kechagias, Emma Nilsson, Fredrik Rorsman and Annika Bergquist. Statistical analysis: Catarina Lindqvist and Michael Ingre. Analysis and interpretation of data and critical revision: All. Drafting of manuscript: Catarina Lindqvist.

## Ethics Statement

Informed written consent was obtained from all patients. This study was conducted in accordance with the Helsinki Declaration and was approved by the Swedish Ethical Review Authority 2018/2462‐31, Dnr 2019‐00611, 2020‐03414 and 2020‐00471 and by the Swedish Medical Products Agency (EudraCT 2018‐200 814‐39). The ClinicalTrials.gov identifier for PiSCATIN is NCT04133792.

## Conflicts of Interest

The authors declare no conflicts of interest.

## Supporting information


Data S1.

## Data Availability

The data that support the findings of this study are available from the corresponding author upon reasonable request.
