# Supplementary material for: Dietary Habits of Individuals With Primary Sclerosing Cholangitis—Poor Fat‐Soluble Vitamin Intake and Dietary Quality
Source: Liver Int. 2024 Nov 27;45(4):e16182. doi: 10.1111/liv.16182 (PMC11907217; doi:10.1111/liv.16182)
Supplement: Supplementary file 1 — Data S1. [file LIV-45-0-s001.docx]

## Supplementary materials

**Table S1**. Dietary intake levels of micronutrients by sex

| **Vitamins and minerals** | **Average requirement NNR 2023** | **All (n=120)** | **Female (n= 33)** | **Male (n=87)** | | **N (%) <AR NNR 2023 all/female/male** | |  |
| --- | --- | --- | --- | --- | --- | --- | --- | --- |
| Thiamine B1 (mg) | *AR 0.65/0.75* | 1.2 (0.8), 1.2-1.4 | 1.0 (0.5), 1.0-1.2 | 1.3 (0.7), 1.3-1.5 | | 8(7)/1(3)/7(8) | |  |
| Niacin B3 (NE) | AR 12/15 | 16.6 (8.1), 16.3-18.8 | 13.7 (8.0), 12.4-15.9 | 17.7 (7.8), 17.3-20.3 | | 36 (31)/11 (34)/25 (29) | |  |
| Riboflavin B2 (mg) | *AR 1.3* | 1.7 (0.9), 1.7-2.0 | 1.4 (0.7), 1.3-1.7 | 1.8 (0.9), 1.7-2.1 | | 30(25)/12(38)/18(21) | |  |
| Folate B9 (ug) | *AR 250* | 291 (173), 302-354 | 266 (118), 254-316 | 299 (211), 310-377 | | 39 (33)/13 (41)/26 (30) | |  |
| Vitamin B12 (ug) | *Provisional AR 3.2* | 5.3 (3.1), 5.2-6.1 | 4.8 (2.4), 4.0-5.1 | 5.5 (3.1), 5.4-6.5 | | 17(14)/7(22)/10(12) | |  |
| Vitamin B6 (mg) | *AR 1.3/1.5* | 1.7 (1.0), 1.7-2.0 | 1.4 (0.6), 1.3-1.6 | 1.9 (1.0), 1.8-2.1 | | 40 (34)13(40)/27(23) | |  |
| Vitamin C (mg) | *AR 75/90* | 78.6 (59.6), 77.3-94.9 | 76.0 (45.3), 70.0-97.0 | 83.4 (67.6), 76.6-99.2 | | 65(55)/16(50)/49(60) | |  |
| Vitamin A (RE) | *AR 540/630* | 682 (468), 698-843 | 601 (336), 551-742 | 753 (511), 712-890 | | 47(40)/12(38)/35(41) | |  |
| Vitamin D (ug) | AR 7.5 | 6.6 (3.6), 6.7-8.1 | 6.0 (3.6), 5.1-7.1 | 7.0 (3.9), 7.0-8.6 | | 70(59)/24(75)/46(54) | |  |
| Vitamin E (mg) | *provisional AR 8/9 a-tocopherol* | 8.9 (5.5), 9.1-10.9 | 7.9 (3.9), 7.5-10.1 | 9.3 (5.8), 9.3-11.5 | | 58 (49)/19(59)/39(45) | |  |
| Vitamin K (ug) | *provisional AR 50/60* | 29.6 (23.9), 31.2-39.6 | 33.1 (18.1), 30.1-39.5 | 28.0 (28.8), 30.2-40.0 | | 105 (89)/28(88)/77(90) | |  |
| Calcium (mg) | *AR 750* | 1060 (612), 1080-1328 | 906 (595), 859-1189 | 1106 (696), 1090-1375 | | 24(20)/9(28)/15(17) | |  |
| Phosphorus (mg) | *provisional AR 420* | 1377 (767), 1407-1657 | 1134 (545), 1113-1397 | 1470 (848), 1459-1761 | | 0(0) | |  |
| Potassium (g) | *provisional AR 2.8* | 3.0 (1.4), 2.9-3.3 | 2.5 (1.3), 2.4-2.9 | 3.0 (1.4), 3.0-3.5 | | 51(43)/21(66)/30(35) | |  |
| Magnesium (mg) | *provisional AR 240/280* | 310 (167), 314-364 | 274 (96), 256-307 | 323 (206), 327-392 | | 39(33)/11(34)/28 (33) | |  |
| Iron (mg) | *AR 9/7* | 8.8 (5.6), 9.1-10.8 | 7.8 (4.2), 7.1-9.0 | 10.0 (6.6), 9.6-11.8 | | 42(36)22(69)/20(23) | |  |
| Zinc (mg) | *AR 8/11* | 9.4 (6.1), 9.9-11.6 | 8.2 (3.3), 7.7-9.5 | 10.4 (6.2), 10.3-12.5 | | 62(53)15(47)/47(55) | |  |
| Sodium (g) | *AI 1.5, max 2.3* | 2.3 (1.1), 2.4-2.8 | 2.0 (0.7), 1.8-2.3 | 2.6 (1.2), 2.5-3.0 | | 13(11)/5(16)/8(9) | |  |
| Iodin (ug) | *provisional AR 120* | 111 (72), 115-137 | 101 (53), 87-115 | 120 (86), 121-148 | | 66(56)/23(72)/43(59) | |  |
| Selenium (ug) | *provisional AR 60/70* | 43.0 (25.8), 44.7-52.8 | 41.3 (15.7), 35.3-45.2 | 45.1 (33.4), 46.6-7.0 | | 96(81)/29(91)/67(78) | |  |
| Median (IQR), 95% CI. Mg; milligram, g; gram, ug; microgram, AR, average requirement, | | | | |  | |  | |

**Table S2**. Dietary intake levels of micronutrients grouped by IBD

| **Vitamins and minerals** | **NNR 2023** | **All (n=120)** | **Non-IBD (n= 28)** | **PSC-IBD (n=92)** | **P** | **N (%) <AR 2023 all/non-IBD/PSC-IBD** |
| --- | --- | --- | --- | --- | --- | --- |
| Thiamine B1 (mg) | *AR 0.65/0.75* | 1.2 (0.8), 1.2-1.4 | 1.2 (0.8), 1.1-1.8 | 1.1 (0.7), 1.2-1.4 | 0.507 | 8(7)/2(7)/6(7) |
| Niacin B3 (NE) | AR 12/15 | 16.6 (8.1), 16.3-18.8 | 17.1 (6.6), 15.7-20.5 | 16.0 (8.6), 16.0-18.9 | 0.531 | 36 (31/8(29)/29(32) |
| Riboflavin B2 (mg) | *AR 1.3* | 1.7 (0.9), 1.7-2.0 | 1.8 (1.2), 1.5-2.2 | 1.7 (0.9), 1.6-2.0 | 0.738 | 8 (29)/23(25) |
| Folate B9 (ug) | *AR 250* | 291 (173), 302-354 | 293 (224), 289-435 | 289 (170), 291-344 | 0.479 | 9 (32)/31 (34) |
| Vitamin B12 (ug) | *Provisional AR 3.2* | 5.3 (3.1), 5.2-6.1 | 5.0 (3.9), 4.5-6.1 | 5.3 (3.0), 5.2-6.3 | 0.594 | 6(21)/11(12) |
| Vitamin B6 (mg) | *AR 1.3/1.5* | 1.7 (1.0), 1.7-2.0 | 1.8 (0.9), 1.6-2.2 | 1.7 (1.0), 1.7-1.9 | 0.673 | 8(29)/33(36) |
| Vitamin C (mg) | *AR 75/90* | 78.6 (59.6), 77.3-94.9 | 87.3 (84.6), 81.5-119.6 | 71.5 (57.9), 71.8-91.7 | *0.058* | 14(50)/53(58) |
| Vitamin A (RE) | *AR 540/630* | 682 (468), 698-843 | 681 (355), 593-863 | 682 (524), 697-870 | 0.700 | 11(39)/36(39) |
| Vitamin D (ug) | AR 7.5 | 6.6 (3.6), 6.7-8.1 | 6.1 (3.8), 5.6-7.9 | 6.9 (3.8), 6.8-8.4 | 0.423 | 17 (61)/54 (59) |
| Vitamin E (mg) | *provisional AR 8/9 a-tocopherol* | 8.9 (5.5), 9.1-10.9 | 8.3 (5.9), 7.7-12.3 | 9.0 (5.3), 9.1-11.0 | 0.515 | 14(50)/45(49 |
| Vitamin K (ug) | *provisional AR 50/60* | 29.6 (23.9), 31.2-39.6 | 29.4 (27.1), 27.4-48.2 | 29.6 (22.1), 30.3-37.7 | 0.814 | 24(86)/83(90) |
| Calcium (mg) | *AR 750* | 1060 (612), 1080-1328 | 1126 (808), 913-1362 | 1050 (585), 1076-1373 | 0.682 | 9 (32)/15(16) |
| Phosphorus (mg) | *provisional AR 420* | 1377 (767), 1407-1657 | 1317 (863), 1220-1790 | 1404 (735), 1400-1681 | 0.507 | 0(0)/0(0) |
| Potassium (g) | *provisional AR 2.8* | 3.0 (1.4), 2.9-3.3 | 2.9 (2.0), 2.8-3.6 | 2.9 (1.3), 2.9-3.3 | 0.607 | 13 (46)/39 (42) |
| Magnesium (mg) | *provisional AR 240/280* | 310 (167), 314-364 | 299 (205), 289-425 | 311 (155), 308-360 | 0.970 | 10(36)/29(32) |
| Iron (mg) | *AR 9/7* | 8.8 (5.6), 9.1-10.8 | 9.1 (4.8), 8.5-13.0 | 8.8 (6.3), 8.8-10.6 | 0.611 | 7(25)/37(40) |
| Zinc (mg) | *AR 8/11* | 9.4 (6.1), 9.9-11.6 | 8.8 (5.0), 8.3-12.7 | 9.6 (5.9), 9.8-11.8 | 0.339 | 16(57)/47(51) |
| Iodin (ug) | *provisional AR 120* | 111 (72), 115-137 | 114 (91), 97-151 | 109 (71), 115-138 | 0.637 | 15 (54)/52(57) |
| Selenium (ug) | *provisional AR 60/70* | 43.0 (25.8), 44.7-52.8 | 42.3 (32.3), 40.6-60.4 | 43.0 (25.7), 43.8-52.7 | 0.877 | 22(79)/75(82) |
| Median (IQR), 95% CI. NNR 2023 ; Nordic Nutrition Recommendations 2023, Mg; milligram, g; gram, ug; microgram, AR, average requirement, | | | |  |  |  |

**Figure S1.** Reported intake of alcoholic beverages, units/week in the study cohort.
